# The role of physical activity promoting thinking skills and emotional behavior of preschool children

**DOI:** 10.1186/s41155-022-00223-1

**Published:** 2022-08-01

**Authors:** Changwei Wang

**Affiliations:** Institute of Physical Education, Ningxia University, Yinchuan, China

**Keywords:** Critical thinking, Emotional development, Imagination, Preschool age, Regular activity

## Abstract

**Objectives:**

Physical activity is critical, not only for the normal growth and development of children, but also for emotional and social behavior. The purpose of the article is to determine the relationship between physical education and social and emotional development of preschool children.

**Methods:**

The study involved 366 children (188 boys and 178 girls) at the ages of 5 (*N* = 191) and 6 (*N* = 174), who study in public kindergartens in Beijing (China). Within 3 months, additional physical education and fitness classes were held. Before and after the study, a test was conducted: Ages & Stages Questionnaires: Social-Emotional (ASQ:SE), which was completed by the parents. The research process did not affect the performance or development of children participants.

**Results:**

As a result of the study, the main regularities of the influence of physical education on social and emotional behavior of children were established. Based on the results of the study, it was determined that there is a positive correlation between age, physical education, and social-emotional behavior (*r* +—= 0.668).

**Conclusion:**

Gender differences are not statistically significant when it comes to physical activity’s effect on social and emotional behavior (*p*-value = 0.004). The results can be applied to programs for the prevention of psychosocial and social-emotional development delays of children in kindergartens.

## Introduction

At present, in the system of scientific knowledge, great attention is paid to the process of physical education of preschool children. At the same time, innovative programs are being developed and new technologies are being introduced (Gordon et al., 2013). Regular physical activity during childhood is not only important for maintaining a healthy body weight, but also brings many other physiological and psychosocial benefits (Frank et al., 2018). There has long been agreement that regular vigorous physical activity provides psychological benefits such as reduced symptoms of depression, the creation of more positive mood states, reduced levels of anxiety, and increased self-esteem in both adults and adolescents, but there is not much information on such effects in young children (Timmons et al., 2007).

Performing physical exercises is accompanied by intense emotional experiences caused by the struggle for the best result. In the process of physical education, the child masters a variety of thinking skills (Mutohir et al., 2019). Unfortunately, the number of children not participating in adequate physical activity continues to be a concern worldwide. The decisive aspect of physical education is the purposeful formation of a conscious, based on deep knowledge and beliefs, motivation of physical training, a stable habit of constantly taking care of one’s health, and instilling skills in organizing daily physical activity (Kain et al., 2017). In modern society, there is an underestimation of the role of physical education in the formation of the integral personality (Rodriguez-Ayllon et al., 2019). Lack of motor activity, muscle weakness, and limited motor skills and abilities lead not only to a child’s loss of physical health, but also to the impossibility of the full-fledged formation of his/her intellectual and emotional components (Cairney et al., 2019). It is very important for preschoolers to engage in any high-intensity physical activity for at least 1 h a day. Up to 6 years old, their body grows every day (Roscoe et al., 2019). Physical activity improves their overall health and is essential for developing healthy habits for life. In addition, physical activity has several benefits for children. It makes them realize the importance of working together, meeting new people, and developing thinking skills (Gao et al., 2019). Physical activity includes physical exercises, walks outside, outdoor games, and training. Outdoor games are the most accessible and effective method of influencing a child. The advantage of outdoor games over sole exercises is that a game is always associated with initiative, individual imagination and requires independence to achieve a game result and responsibility to a team (Schembri et al., 2019). A healthy and active child is automatically self-confident and dignified. Physical activity improves the overall level of concentration, allows children to cope better with anxiety and stress, and improves motor skills and brain development (Burns et al., 2017; Lubans et al., 2016). Children who do not receive adequate instruction and practice in motor skills may have a delay in their development (Stagnitti et al., 2011). Social-emotional development of preschoolers is one of the basic components of mental health. Physical culture means can be used to stabilize the emotional response when interacting with the environment (Denham et al., 2015). Based on a literature review, a child’s social competence was defined as the ability to establish and maintain social contacts in the process of interaction based on a positive attitude towards oneself, and emotional competence is the ability to experience emotions, understand one’s own emotions and the emotions of other people, and regulate their expression (Franco et al., 2017). Research on early childhood points to the concept of emotional competence, skills, and behaviors in children associated with emotions such as expression, awareness, labeling, and understanding of emotions based on emotional facial expressions and contextual cues (Kılıç, 2015). The concept of social-emotional competence (SEC) seems the most integrative as a set of additional abilities:

1. Self-awareness, which describes the knowledge of one’s strengths and weaknesses and distinction of emotions.
2. Empathy—the ability to recognize, understand, and compassionate the feelings of another and the desire to influence them positively.
3. Motivation indicates the ability to stay involved in an activity, even if it is difficult or the previous experience was partially related to failure (Costa et al., 2020).
4. Self-regulation, which is the conscious control of impulsive and inappropriate reactions and inappropriate expressions of emotion (Weare, 2004).
5. Thinking skills that include a wide range of knowledge, abilities, and competencies, including the ability to develop and maintain healthy relationships, participate in social activities, and interact with people correctly (Aubrey et al., 2012).

In addition, research highlights the need to use critical thinking to predict positive gains such as adjusting to school and developing positive attitudes, academic performance, attachment, and social behavior (Fernández-Santín & Feliu-Torruella, 2020). In addition to the personal characteristics of preschoolers, external factors (family, educator, country) influence the formation of critical thinking (Breaux et al., 2016).

Teachers need to have knowledge about thinking skills, know how to teach those skills through activities including thinking skills, become aware of the difficulties that learners may encounter, and develop preventive methods for those difficulties in order to enable learners to become effective thinkers rather than transferring knowledge to their learners (Akinoglu & Karsantik, 2016).

Definitely, it is difficult for a child to go through the entire process of socialization on his/her own. The role of parents, teachers, and the environment is great for this. An active game can help in this—an activity that is close to children in perception and images. It carries a great pedagogical value (Domitrovich et al., 2007). Topics related to the influence of physical activity on the psychoemotional state of preschool children had been previously studied. At the same time, there are few studies devoted to the analysis of the connection between physical education and social and emotional development of preschoolers. For this reason, this research is considered relevant and innovative.

The significant influence of physical activity and motor experience on the cognition of the world around young children has recently been associated with the effects of neurotrophic brain plasticity (Lippi et al., 2020; Miskolczi et al., 2019). At the age of up to 5 years, the activation of neuron growth is largely stimulated by motor activity. However, as evidenced by a number of studies, from an older age, social factors of communication and socialization processes begin to increasingly significantly dominate the processes of formation of a child’s cognitive functions (Miskolczi et al., 2019; Ploughman, 2008). However, studies on the elderly suggest that targeted and increased physical activity can trigger the mechanisms of neurotrophic plasticity at any age (Cho & Roh, 2019). Therefore, the main question of the study presented here is whether it is possible only by increasing the amount of ordinary physical activity of the type of movements familiar and studied by the child to enhance the development of cognitive capabilities, in particular, critical thinking.

In accordance with the purpose of this study, the following tasks were set:

1. Determining the relationship between physical education classes and critical thinking
2. Identifying social and emotional problems of young children before and after intensive physical education classes
3. Determining the impact of gender and age differences among study participants on physical activity

## Materials and methods

### Ethical issues

Participation in the study was voluntary and with the parents’ permission who completed the consent form. The research process did not affect the performance or development of children participants. No personal data of the participants were collected, stored, or used during the study. The author declares that the work is written with due consideration of ethical standards. The study was conducted in accordance with the ethical principles approved by the author’s university (Protocol No 3 of 15.02.2019).

### Participants

The study involved 366 children (188 boys and 178 girls) aged 5 (*N* = 191) and 6 (*N* = 174) years old who study in public kindergartens in Beijing (China). Other 113 children were excluded due to lack of parental consent for the study.

Participants were selected at random. An invitation to participate, guarantees of anonymity, and an explanation of the reasons and objectives of the study were provided to the parents of the participating children. Information about the child participants was voluntarily provided by their parents after their consent to participate in the study.

### Intervention

The research was carried out for 2 months. Children took part in rhythmic and physical education classes, which, in contrast to the adopted state educational program for preschool institutions, were held every day. Before and after the study, the tests were conducted.

As a basis for the intensification of physical education, the training program already familiar and adapted by these children, practiced in their kindergartens, was taken. This program is common to public kindergartens in Beijing, which eliminates the need for additional education or training for the staff training children. This program uses 3 days a week for 40 min of classes; during the lesson, rhythmic exercises are used to the music and at the expense of the teacher, as well as gymnastic exercises, including simple squats; bends; turns; warm-up of the joints of the arms and legs with circular movements of small, medium, and increasing amplitude; various types of running in a circle; and jumping in place. Rhythmic exercises include dancing or a series of isolated simple dance and gymnastic movements aimed at developing a sense of rhythm, increasing coordination and accuracy of orientation in space. The exercises are performed by all children at the same time under the guidance of a teacher. The session is usually broken down into 2 or 3 sequences, separated by rest and free play, breathing exercises, or quiet sitting and relaxation.

The intensification program (intervention) consisted of three significant changes. (1) The lessons described above were necessarily carried out every day. (2) As breaks between sequences during classes, only purposeful breathing exercises or deliberate relaxation while sitting under the guidance of a trainer was used. (3) After the end of physical classes, children were necessarily given a break of at least 30 min for games, distraction, personal activities, and recovery.

These changes were selected on the basis of the recommendations of doctors supervising the children in these kindergartens in order to maintain health and not exceed the permissible loads on the body of children.

The increase in cognitive abilities was equally dependent on the increase in the amount of physical activity and social interaction of children, because the classes were conducted in a group. The significance of the impact of physical movement should be assessed as a more significant factor, since the amount and quality of social interaction for children did not actually change over this time (Miskolczi et al., 2019). The need to take into account the effects of neurotrophic plasticity in this case is reduced, because at the age of 5 years, their role naturally decreases, and the role of motor activity and social interaction increases (Lippi et al., 2020).

### Procedure

Ages & Stages Questionnaires: Social-Emotional (ASQ:SE) with sets of questions for children 5 and 6 years old (Squires et al., 2002) was used. The questionnaire contains 33 questions, each of which characterizes a specific aspect of a child’s behavior: self-regulation, compliance, communication, adaptive behaviors, autonomy, affect, and interaction with people (Squires et al., 2002). The following options were offered: “most of the time” = 0 points, “sometimes” = 5 points, “rarely or never” = 10 points, and “checked concern” = 5 additional points. In ASQ-SE, lower scores indicate more positive results. If the child’s overall score exceeds the threshold of 70, this means that additional mental health assessment may be required. The more points the test gets, the higher the risk of delays in the emotional and social development of the child it shows (Squires et al., 2002). The study used the mean values for the control and experimental groups.

The Cornell Critical Thinking Test (CCTT) is one of the many multiple-choice tests with validated questions that have been reported to measure general critical thinking (CT) ability. Level X is a 71-item, multiple-choice test for students in grades 5–12 + (Ennis et al., 2005; Leach et al., 2020). It may be administered as a 50-min timed or as an untimed evaluation. There are three possible answers. The max score is 71.

These tests are used because of their widespread use in the practice of social workers, child psychologists, and academic practice. Their validity and applicability have been repeatedly confirmed, including in the framework of empirical studies (Aizikovitsh-Udi & Cheng, 2015; Ennis, 2018; Heo & Squires, 2012; Kwan & Wong, 2015). The test has numerous national adaptations (Alvarez-Nuñez et al., 2020; Chen et al., 2017).

Each of the two tests used in the study was conducted in the present but without the active participation of the parents, which provided calmness, confidence, and support for the children during the test. The tests were carried out on separate days so as not to overwhelm the children. Before the start of the test, the children were introduced to it and explained in detail the meaning of each question, if any questions arose. On average, tests took from 30 min to 1 h; all questionnaires were successfully completed by participants; no questionnaire was subsequently rejected for any reason.

The survey was conducted with the participation of researchers and a number of student volunteers who have experience in interacting with children of the appropriate age. The questionnaires were completed by researchers and volunteers according to the children’s responses. Before entering the answer to the questionnaire, the researchers and volunteers had to make sure that the child correctly understood the content of the question on the questionnaire. If the child needed a short break for rest, then he/she definitely received it.

### Data analysis

Correlation analysis (Pearson) was used to study the relationship between age and the level of critical thinking of children. Data were analyzed using SPSS Statistics version 10. For pre- and post-ASQ:SE test results, the mean of the test and the sample standard deviation were examined. Student’s *t*-test was also used (*p* < 0.05) to check the equality of the mean values in the two samples. A correlation analysis and Student’s *t*-test were conducted to compare the pre-test and post-test results for the groups of boys and girls separately and for children aged 5 and 6 (Table 3). Thus, the null hypothesis was tested that the use of physical culture does not affect the critical thinking of children. The margin of error was 2.9%. Some of the questionnaires were not filled out correctly (for example, some respondents did not answer all the questions of the questionnaire).

### Research limitation

The studies were carried out among children who are raised in urban kindergartens and did not take into account the countryside. Social status and racial differences were not taken into account. The study also covers a group of preschoolers between 5 and 6 years old, which limits the ability to draw conclusions for other groups of children, since the level of development and social-emotional problems and thinking skills at different levels of child development differs.

## Results

According to the research results, the average value of critical thinking does not exceed the threshold value of 70 points. A detailed analysis is presented in Table 1.

**Table 1:** Statistical evaluation results of the Cornell Critical Thinking Test (CCTT) for pre- and post-test

	Pre-test	Post-test
Average test value	63	47
Standard deviation	1.34	0.93
*p*-value		0.049
Pearson correlation		0.675

*p* < 0.05

The results show (Table 1) that there is a statistical significance (*p*-value = 0.049) between physical education and the level of critical thinking of 5- and 6-year-old children. After completing an enhanced course of physical education, the average value of the post-test decreased by 16 points. This result goes far beyond the margin of error (standard deviation in the sample). A decrease in the test indicator indicates a decrease in the risks of delaying the critical thinking development of children by means of physical education. According to the test results, there is a positive and strong correlation (0.675) between the Cornell Critical Thinking Test and physical activity. It should be recognized that well-planned physical activity has a positive effect on the development of critical thinking.

Table 2 presents the characteristics of social-emotional problems before and after the experiment.

**Table 2:** Statistical evaluation results of ASQ:SE for pre- and post-tests (for each test component)

Indicator	Pre-test, *N* = 366, M (SD)	Post-test, *N* = 366, M (SD)	*r* (Pearson correlation)	*p*-value
Self-regulation	9.6 (0.7)	7.3 (0.2)	0.634	0.029
Compliance	9.1 (0.4)	6.2 (0.4)	0.65	0.037
Communication	10.1 (0.5)	5.6 (0.7)	0.601	0.045
Adaptive behaviors	10.4 (0.7)	5.3 (0.6)	0.643	0.047
Autonomy	8.6 (0.4)	5.4 (0.1)	0.716	0.018
Affect	8.4 (0.3)	6.3 (0.3)	0.699	0.021
Interaction with people	11.8 (0.4)	5.2 (0.6)	0.658	0.051

*p* < 0.05

In general, the results show statistically significant indicators for each characteristic. Besides, the data obtained indicate that after 3 months of physical education according to the enhanced program, the performance of children in all main aspects of emotional and social criteria increased in a positive direction. Physical activity has the most significant effect on interaction with people (*p* < 0.051). In addition, according to the results, physical education improves communication skills (*p* < 0.045) and promotes better adaptability to external factors (*p* < 0.047). Sports activities have the least impact on the autonomy of children, *p* < 0.028, while the indicator is within the limits of statistical significance. In addition, it should be noted that before physical education, children had more perceptible problems with communication (10.1), adaptation to the external environment (10.4), and interconnection with people (11.8), as evidenced by a fairly high average score for these indicators. There are also less significant problems, such as shifting attention from one activity to another, the ability to self-organize, and the ability to play alone.

CCTT test results by age and gender are presented in Table 3.

**Table 3:** Statistical evaluation results for the gender and age criteria

		Pre-test	Post-test	Pearson correlation	Student’s *t-*test *p*-value
Gender					
Boys	M (SD)	62.2 (6.26)	48.1 (2.1)	0.675	0.004
Girls	M (SD)	61.3 (1.46)	47.3 (1.9)	0.654	0.014
Age (years)					
5	M (SD)	61.2 (1.63)	46.3 (2.2)	0.645	0.022
6	M (SD)	56.1 (1.62)	41.1 (2.3)	0.701	0.003

The *p*-value for age and gender categories was determined for the post-test. The results of the study by gender and age generally repeat the CCTT indicators for the entire sample.

Gender differences are not statistically significant when it comes to the physical activity effect (*p*-value = 0.004). In turn, age is statistically significant (*p*-value = 0.022) for the critical thinking of children. Older children have a better level of critical thinking, as evidenced by the results of the post-test. The indicator for 6-year-old children is 5.2 points lower than for 5-year-olds. Regardless of gender, improvement in critical thinking correlates with increased physical education. At the same time, the correlation is not significantly different for girls (0.654) and boys (0.659).

There is also a fairly strong correlation between the pre-test and post-test results for children 5 and 6 years old. However, for children aged 6 years, the correlation is stronger (0.701) than for children aged 5 (0.645). What is the reason for this phenomenon is a question for further research.

## Discussion

This study showed that physical activity has a positive effect on social and emotional well-being. It should be noted that a child’s age influences critical thinking development. Research has shown that indicators of critical thinking improve for 6-year-olds. Overall, this study confirms the positive impact of physical activity on the emotional and social states of children. The post-test results improved by an average of 4 points on all criteria, which is statistically significant since it goes beyond the margin of error (standard deviation in the sample). This indicator allows one to conclude that children who were immersed in an environment of increased physical activity through daily physical education, outdoor games, and rhythmic improved their mood and became happier. In addition, preschoolers have ceased to conflict and throw tantrums. In accordance with the results of the test on the criterion of interaction with people, after a long period of intensive physical education, children prefer group games and react more positively to new people. The results of the communication characteristic in the post-test decreased by almost 5 points, which testifies to the fact that due to physical activity, children can more easily make new acquaintances and become more sociable. The results of the post-test showed that children became more independent and responsible.

The current study found that gender differences are not statistically significant when it comes to physical activity’s effect on the critical thinking of boys and girls, unlike age which has a significant difference. There is a positive correlation between age, physical activity, and critical thinking (*r* +—= 0.668). The results of the study do not directly resonate with the work of other authors but receive additional confirmation in a number of studies. Similar studies showed that active children were more energetic and restless, less inhibited, less compliant, less shy, more assertive, more competitive, and more manipulative than their less active peers (Rodriguez-Ayllon et al., 2019). Scientists note that physical activity and outdoor games increase the level of critical thinking (Hyndman et al., 2020; Ip et al., 2016). In contrast to the present study, some studies have noted gender differences. Namely, emotional and psychological problems in boys are easier to observe since they are more pronounced and therefore more reported by their parents (Maguire et al., 2016). Research on the emotional development of children shows that teachers play an important role in recognizing and regulating children’s emotions; teachers are considered quite important role models in terms of how children express emotions (Roberts et al., 2016). According to the results of a survey of Chinese physical culture specialists, 29.6% of respondents believe that physical education is a process of mastering the means and methods of physical culture, 27.2% that it is the education of moral and volitional qualities, and only 2.3% that it is a training process. The majority of respondents, 40.9%, agree that the process of physical education is nothing more than a process of education, and, in the opinion of the respondents, employees of the relevant state administration should determine the directions and content of physical education (42.2%), as well as scientists (25.8%) and teachers (32%). One of the factors influencing the effectiveness of this process is determined by a complex of pedagogical conditions—the enrichment of the basic forms and methods of physical education (Truelove et al., 2020). Several studies (meta-analysis) have found that physical activity may be associated with an increased cognitive performance during childhood. A positive association was found between physical activity and cognitive function, which includes motor skills, IQ, academic achievement, verbal and math tests, developmental level, and critical thinking in school-aged children (Hujar & Matthews, 2021; Yates & Twigg, 2017).

Some studies recommend increasing physical activity and play time for preschool children (Chaddock-Heyman et al., 2013). The development of the emotional sphere of older preschool children in the process of communicating with peers is determined by the type of situational communication and the prevailing experience of communicating with peers, the ability to choose constructive ways of solving problem situations in the process of communication (Scrimgeour et al., 2016).

### Conclusion

The problem of the social and emotional development of children is relevant at all times. Teachers need to know what points to pay attention to when raising children and what means to use in this case. The literature analysis showed that the role of physical education in the formation of critical thinking is underestimated. The aim of the study was to try to establish a link between physical education, social-emotional behavior, and critical thinking of preschool children using the example of Beijing (China). Based on the results of the study, it was determined that there is a positive correlation between age, physical education, and critical thinking (*r* +—= 0.668). It was found that gender does not have a statistical significance when it comes to the effect of physical activity on the critical thinking of children (*p*-value21 = 0.004). In turn, the age of a child is important since statistically significant differences were found (*p*-value = 0.022). Also, it was determined that physical education has a positive effect on the emotional and social behavior of preschoolers. A practically significant contribution and novelty of this study is the provision of experience of a significant improvement in the results of critical thinking tests in children only with the intensification of already existing and tested programs of physical development, taking into account proper rest and rhythm of classes, even without using specialized additional methods of physical development.

## Data Availability

Data will be available on request.
